# Acceptance of digital phenotyping linked to a digital pill system to measure PrEP adherence among men who have sex with men with substance use

**DOI:** 10.1371/journal.pdig.0000457

**Published:** 2024-02-22

**Authors:** Hannah Albrechta, Georgia R. Goodman, Elizabeth Oginni, Yassir Mohamed, Krishna Venkatasubramanian, Arlen Dumas, Stephanie Carreiro, Jasper S. Lee, Tiffany R. Glynn, Conall O’Cleirigh, Kenneth H. Mayer, Celia B. Fisher, Peter R. Chai

**Affiliations:** 1 The Fenway Institute, Fenway Health, Boston, Massachusetts, United States of America; 2 Department of Emergency Medicine, Brigham and Women’s Hospital, Boston, Massachusetts, United States of America; 3 Department of Psychiatry, Massachusetts General Hospital, Boston, Massachusetts, United States of America; 4 Department of Computer Science and Statistics, The University of Rhode Island, Kingston, Rhode Island, United States of America; 5 Department of Emergency Medicine, University of Massachusetts Chan Medical School; 6 Department of Medicine, Beth Israel Deaconess Medical Center, Boston, Massachusetts, United States of America; 7 Center for Ethics Education, Fordham University, New York City, New York, United States of America; 8 Department of Psychosocial Oncology and Palliative Care, Dana Farber Cancer Institute, Boston, Massachusetts, United States of America; 9 The Koch Institute for Integrated Cancer Research, Massachusetts Institute of Technology, Cambridge, Massachusetts, United States of America; Iran University of Medical Sciences, IRAN (ISLAMIC REPUBLIC OF)

## Abstract

Once-daily oral HIV pre-exposure prophylaxis (PrEP) is an effective strategy to prevent HIV, but is highly dependent on adherence. Men who have sex with men (MSM) who use substances face unique challenges maintaining PrEP adherence. Digital pill systems (DPS) allow for real-time adherence measurement through ingestible sensors. Integration of DPS technology with other digital health tools, such as digital phenotyping, may improve understanding of nonadherence triggers and development of personalized adherence interventions based on ingestion behavior. This study explored the willingness of MSM with substance use to share digital phenotypic data and interact with ancillary systems in the context of DPS-measured PrEP adherence. Adult MSM on PrEP with substance use were recruited through a social networking app. Participants were introduced to DPS technology and completed an assessment to measure willingness to participate in DPS-based PrEP adherence research, contribute digital phenotyping data, and interact with ancillary systems in the context of DPS-based research. Medical mistrust, daily worry about PrEP adherence, and substance use were also assessed. Participants who identified as cisgender male and were willing to participate in DPS-based research (N = 131) were included in this subsample analysis. Most were White (76.3%) and non-Hispanic (77.9%). Participants who reported daily PrEP adherence worry had 3.7 times greater odds (95% CI: 1.03, 13.4) of willingness to share biometric data via a wearable device paired to the DPS. Participants with daily PrEP adherence worry were more likely to be willing to share smartphone data (p = 0.006) and receive text messages surrounding their daily activities (p = 0.003), compared to those with less worry. MSM with substance use disorder, who worried about PrEP adherence, were willing to use DPS technology and share data required for digital phenotyping in the context of PrEP adherence measurement. Efforts to address medical mistrust can increase advantages of this technology for HIV prevention.

## Author summary

Oral medications for HIV pre-exposure prophylaxis (PrEP) are highly efficacious in preventing HIV infection, but efficacy is closely linked with adherence. Despite the availability of PrEP, measuring adherence and responding to nonadherence events remains difficult. One possible strategy to measure PrEP adherence is using a digital pill system (DPS) that activates directly in the stomach and reports adherence events. Integrating contextual markers like smartphone digital phenotyping may enhance behavioral interventions that leverage DPS adherence data to provide PrEP adherence support. Here, we conducted a survey study through a social networking website to understand perceptions of the DPS and linked digital phenotyping among MSM with substance use on PrEP. We found that the degree of substance use did not mediate willingness to participate in research using digital phenotyping and the DPS. Individuals who worried more about PrEP adherence were more willilng to interact with the DPS and digital phenotyping techniques.

## Introduction

Once-daily oral pre-exposure chemoprophylaxis (PrEP) is highly efficacious in preventing human immunodeficiency virus (HIV) acquisition when adherence is maintained [1]. Following results from multiple clinical trials, tenofovir disoproxil fumarate/emtricitabine (TDF/FTC) was recommended by the World Health Organization (WHO) and the United States (US) Centers for Disease Control and Prevention (CDC) for use as oral PrEP in 2012 [2]. Over the past decade, PrEP has become widely recognized as a key pillar of the strategy to end the HIV epidemic and is recommended for populations at risk of HIV acquisition. Among individuals at risk, men who have sex with men (MSM) experience disproportionate HIV exposure, particularly given common comorbidities of mental health, stigma, and trauma [3]. Additionally, substance use among MSM has been independently associated with an increased risk of HIV acquisition and PrEP nonadherence [4]. Despite the recent success of long-acting injectable cabotegravir as PrEP [5], there remains a need to develop strategies to assess and improve oral PrEP adherence, especially among MSM who may not qualify or be unable to access injectable PrEP.

Given the importance of initiating and maintaining PrEP use for HIV prevention efforts, several tools have been developed to measure adherence [6]. These include both indirect methods that infer medication ingestion events (e.g., self-report, pharmacy refill records, smart pill bottles) and direct methods (e.g., directly observed therapy, video-assisted observed therapy, and measurement of drug levels in biological matrices) [7]. Another tool that allows for direct measurement of adherence is a digital pill system (DPS), which provides confirmation of the presence of an ingested medication in the stomach. The FDA-cleared DPS (etectRx, Gainesville, FL) comprises a standard gelatin capsule with an integrated radiofrequency emitter that over-encapsulates PrEP. Upon ingestion, gastric chloride ions activate the radiofrequency emitter, transmitting a prespecified radiofrequency signal to an off-body wearable device (Reader), which stores and forwards ingestion data to a smartphone app, where DPS users and clinical or research teams can view real-time adherence data [8]. This system can also serve as a platform for the delivery of tailored adherence interventions, which can be directly informed by changes in detected PrEP adherence patterns over time [9].

Previous qualitative work demonstrated that MSM with substance use are accepting of DPS technology, willing to operate it in the real world to measure PrEP adherence, and perceive value in having on-demand access to PrEP adherence data [8,10]. Additionally, a recent study surveyed a national sample of MSM on PrEP who use substances to understand broader perceptions of the DPS and willingness to interact with the system for PrEP adherence measurement [11]. The results were congruent with previous qualitative work demonstrating the willingness of MSM on PrEP who use substances to interact with the DPS. Participants also described an interest in accessing their adherence data on demand, and those with greater worry surrounding their PrEP adherence were statistically significantly more willing to interact with the DPS. The current investigation builds off of previous research by exploring the willingness of MSM who use substances to engage with ancillary devices and systems, and to share smartphone data, in the context of DPS-based research.

One advantage of DPS technology lies in its ability to capture detailed daily patterns of ingestions. Such patterns of medication adherence behavior can form the basis of systems that seek to measure the context in which ingestions occur [12]. The increasing ubiquity of smartphone ownership and use of wearable, health-related devices [13] presents an additional opportunity to collect and leverage passive device data (e.g., battery life, accelerometry, and global positioning system [GPS] data) to identify digital traits that may be indicative of changes in adherence behaviors, such as adherence. Digital phenotyping–the practice of aggregating large amounts of passive smartphone and wearable data–has been demonstrated to indicate exacerbations of mental health and pain among individuals with mental illnesses and acute bony fractures [14–16] and has been used to track and monitor changes in the health status of surgical care patients [17].

Applied to DPS-measured PrEP adherence, digital phenotyping may contribute detailed insights to contextualize ingestion events and potentially anticipate situations in which nonadherence may occur. While the combination of digital phenotyping and DPS-based adherence data has the potential to deliver real-time tailored adherence interventions in the future, this has not yet been tested empirically. Despite demonstrated acceptance of DPS among substance using MSM, the addition of data from ancillary devices like smartphones or wearable devices may be perceived as a further encroachment of privacy, especially among a population that experiences heightened stigma surrounding sexual orientation, substance use and HIV risk. This investigation sought to examine the association between willingness to share digital phenotypic data and interact with ancillary devices and systems in the context PrEP adherence DPS-based research, and daily PrEP worry, medical mistrust, and degree of substance use, among HIV-negative MSM with substance use.

## Methods

### Study design

**Fig 1:**
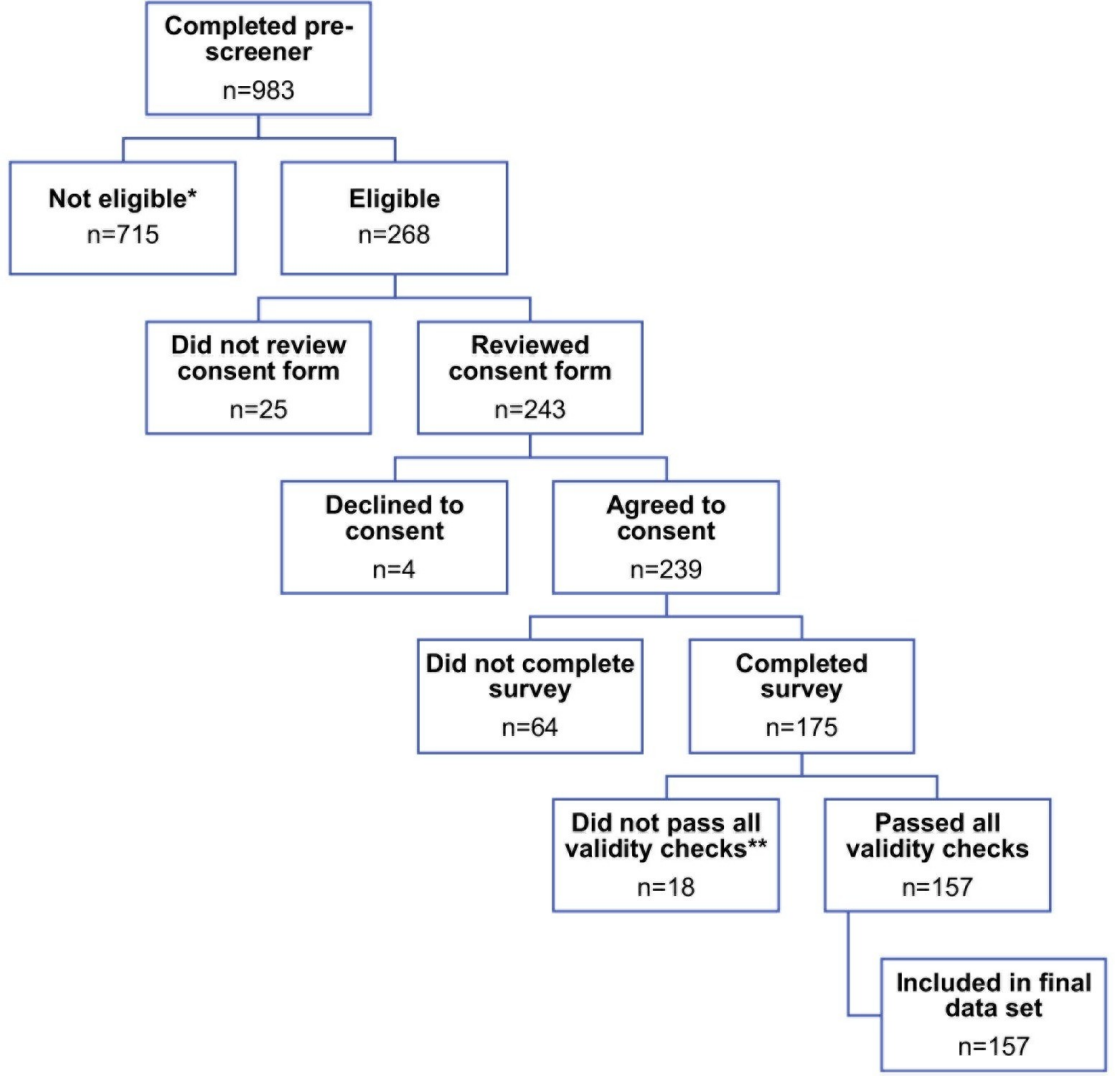
Study design and methods. * Of 715 ineligible individuals, 343 were ineligible for more than one reason. Reasons for ineligibility included: not ≥18 years old (n = 2), not cisgender or transgender male (n = 79), does not have sex with cisgender or transgender males (n = 37), not HIV-negative (n = 101), not on PrEP (n = 367), not sexually active in the last three months (n = 67), and CAGE-AID score <2 (n = 562). ** Of 18 participants who did not pass all validity checks, 1 participant failed to pass more than one validity check. Reasons for not passing all validity checks included: age and date of birth did not match (n = 15), home zip code and home state did not match (n = 2), and IP address did not confirm current location in the US (n = 2).

This was a one-time cross-section online sampling-based survey of a national sample. Please see Fig 1 below for a graphical representation of the study design and methods.

### Participants

The eligibility criteria for the parent study were as follows: (1) 18 years or older; (2) cisgender or transgender MSM; (3) self-reported HIV-negative; (4) currently on PrEP; (5) self-reported sexually active in the past 3 months; (6) score of two or higher on the CAGE Questions Adapted to Include Drug Use (CAGE-AID) [18]; and (7) current user of the Grindr social networking app.

### Procedures

Participants were recruited through an advertisement partnership with Grindr (West Hollywood, CA), a popular social network site that caters to gay, bisexual and transgender people. The study advertisement was delivered to 1,000,000 active US Grindr users via an inbox message, which was active for 24 hours in January 2022. The study advertisement was paid for by the study team via the Fordham University Research Ethics Training Institute (NIH R25DA031608). The study team was composed of cisgender heterosexual and sexual minority people trained in research surrounding technologies and HIV treatment/prevention. No members of the study team have commercial interests in digital pill systems or the digital phenotyping techniques described in this manuscript. Grindr was not involved in the design or conduct of the study or data analysis.

Individuals who clicked on the study advertisement were linked to an eligibility screener via a computer-assisted self-interviewing (CASI) secure platform (Qualtrics, Provo UT), followed by a CAPTCHA validation question. Eligible individuals were presented with a fact sheet containing detailed study information, including a description of the study, study contact information, and an overview of study objectives and potential risks. After independently reviewing the fact sheet, participants documented their informed consent by selecting “I agree to participate.” Participants were provided with the option to download and save the fact sheet for future reference.

Participants completed a cross-sectional quantitative assessment via a computer-assisted self-interviewing (CASI) secure platform (Qualtrics, Provo UT), which lasted approximately 30–60 minutes. The study team conducted several manual validity checks following survey completion (i.e., a confirmed match between age and date of birth, the validity of US home zip code, match between zip code and home state, and IP address indicated location within the US) to confirm eligibility for remuneration. Anonymized survey responses were stored on the secure Qualtrics platform after survey completion, and all validated anonymized datasets were exported, password-protected, and stored on a HIPAA-compliant Dropbox Business folder accessible only to study staff. All study staff were trained in data management and quality assurance protocols prior to the onset of the study.

Of the parent sample (N = 157), only those who reported at least slight willingness to participate in DPS-based research and self-identified as cisgender males were included in the subsample (N = 131). Individuals who self-identified as transgender were excluded from the subsample (n = 6) due to the small sample size and potential for significantly different experiences with the medical system and HIV risk factors, as compared to cisgender MSM. The Fenway Community Health Institutional Review Board (IRB) reviewed and approved all study procedures.

### Measures

The quantitative assessment included an eligibility pre-screener, an overview of the DPS technology–including images of the DPS components, and a video (recorded by PRC) explaining system functionality–followed by survey questions as detailed below.

### Sociodemographics

Participants reported age, race, ethnicity, gender, sexual orientation, education, annual income, and geographic region (i.e., US census region). Participants also indicated their PrEP adherence over an average week in the past three months (i.e., PrEP adherence), and how long they have been taking PrEP (i.e., PrEP duration).

### Willingness to participate in DPS-based research

After viewing a series of informative images and a video explaining how the DPS works, participants were asked to rate their willingness to participate in future DPS-based research studies on a 5-point Likert scale (1 = not at all, 2 = slightly, 3 = moderately, 4 = very, 5 = extremely willing). Those who indicated at least slight willingness to participate in future DPS-based research were included in the final subsample.

### Willingness to contribute digital phenotyping data and interact with ancillary systems in the context of DPS-based research

We assessed participants’ willingness to contribute smartphone data (e.g., geographic location, battery level, text messaging, frequency of use of the app connected to the DPS); self-collected blood work (finger prick) in the context of DPS-based research; share biometric information (e.g., physiologic vital signs) during PrEP use via a wearable device paired to the DPS; and willingness to receive text messages asking about substance use, sexual activity, general daily activities, and location. Participants rated their willingness for each of the above items on a five-point Likert scale (1 = not at all, 2 = slightly, 3 = moderately, 4 = very, 5 = extremely willing), which was then collapsed into two categories for analysis (1 and 2 = “slightly or not willing”; and 3, 4 and 5 = “willing or extremely willing”).

### Medical mistrust

Degree of mistrust in research and medical settings was measured via an adapted, 6-item version of the Group-Based Medical Mistrust Scale (GBMMS), which has been demonstrated as a reliable and valid measure for assessing research mistrust among American adults [19]. The GBMMS is comprised of six questions scored using a 5-point Likert scale (1 = strongly disagree, 5 = strongly agree). Items are summed to calculate a cumulative medical mistrust score, with higher scores indicating greater mistrust (range: 6–25) [19].

### Substance use

As part of the eligibility screener, participants completed the CAGE Questions Adapted to Include Drug Use (CAGE-AID), which has been previously demonstrated as reliable and valid measure [18,20]. The CAGE-AID comprises four yes/no questions about substance use (i.e., perceived need to cut down on substance use, annoyance when substance use is criticized by others, feelings of guilt about substance use, and use of substances first thing in the morning). “Yes” responses are scored as 1 and “No” responses are scored as 0. Items are summed for a total score (possible range: 0–4), with higher total scores indicating greater potentially problematic substance use, and scores ≥2 considered clinically significant. Participants were categorized into three groups based on CAGE-AID score (i.e., 2, 3, and 4).

### Daily PrEP worry

Participants reported their degree of daily worry about PrEP adherence on a single question via a 5-point Likert scale (1 = not at all, 2 = slightly, 3 = moderately, 4 = very, 5 = extremely willing). Responses were collapsed into two categories for analysis (1 and 2 = “slightly or not worried”; and 3, 4 and 5 = “worried or extremely worried”).

### Data analysis

Descriptive statistics were generated for sociodemographic variables. A multivariable logistic regression model was used to measure the association between each of the outcome variables (i.e., willingness to share smartphone data; self-collected blood work in the context of DPS-based research; use a wearable device paired to the DPS to collect biometric information during PrEP use; and to receive text messages asking about substance use, sexual activity, general daily activities, and locations) and independent variables of interest (i.e., daily PrEP worry, medical mistrust (GBMMS), and substance use (CAGE-AID)). A multivariate logistic regression model was used due to medical mistrust confounding the association between the outcome variables and the predicator variable of daily PrEP worry. We also assessed for a potential confounding effect by the following covariates: age, education level, race/ethnicity, PrEP adherence, and PrEP duration. After assessing for a potential confounding effect on the association between the outcomes of interest and independent variables of interest by using Chi-square tests, we determined that the enlisted covariates above did not confound the association between the predictors and outcome variables (p-values > 0.05). Covariates were evenly distributed among the predictor variables. Therefore, the covariates listed above were not included in the model. All analyses were completed using SAS (version 9.4) [21]. The SAS code PROC LOGISTIC was used to conduct the multivariable logistic regression model.

## Results

### Sociodemographics and willingness to participate in future DPS research

Details on the parent sample (N = 157) are reported elsewhere [11]. In this subsample analysis, only individuals who reported at least a slight willingness (1 = not at all, 2 = slightly, 3 = moderately, 4 = very, 5 = extremely willing) to participate in DPS-related research and self-identified as cisgender males were included (N = 131). There were no significant differences in sociodemographic characteristics between participants who indicated they would not be willing to participate in DPS-related research (N = 20) and those who did.

The mean age of the subsample (N = 131) was 36.6 (SD: 12). The majority were White (n = 100, 76.3%), non-Hispanic (n = 102, 77.9%), completed at least some college (n = 121, 92.4%), and reported an annual income of more than $60,000 (n = 77, 58.8%). More than half the sample reported being on PrEP for more than a year (n = 75, 57.3%), with the vast majority of participants self-reporting ≥ 4 doses per week during a typical week (n = 124, 94.7%) (Table 1).

**Table 1:** Sociodemographic characteristics (N = 131).

Variable	n (%)
Age (years)	
Mean (SD)	36.6 (12)
Race	
White	100 (76.3)
African American	5 (3.8)
Asian	5 (3.8)
American Indian or Alaska Native	2 (1.5)
More than one race	15 (11.5)
Other	4 (3.1)
Ethnicity	
Not Hispanic or Latinx	102 (77.9)
Hispanic or Latinx	29 (22.1)
Gender Identity	
Cisgender male	131 (100.0)
Education	
High school degree or some high school	10 (7.6)
College degree or some college	78 (59.5)
Graduate/professional degree or some graduate work	43 (32.8)
Annual Income	
Less than $24,000	22 (16.8)
$24,000 to $29,999	12 (9.2)
$30,000 to $59,999	20 (17.3)
$60,000 or more	77 (58.8)
Geographic Region (in US)	
Midwest	20 (15.3)
Northeast	40 (30.5)
South	47 (35.9)
West	24 (18.3)
PrEP Adherence	
<4 doses per week	7 (5.3)
≥ 4 doses per week	124 (94.7)
PrEP Duration	
Less than 1 month	7 (5.3)
1 to 6 months	31 (23.7)
6 months to 1 year	18 (13.7)
More than 1 year	75 (57.3%)

### Willingness to share biometric information via a wearable device paired to the DPS

There was a statistically significant association between the willingness to use a wearable device to collect biometric information and both daily PrEP worry (p = 0.046) and medical mistrust (p = 0.005). Participants who reported being worried about daily PrEP adherence had 3.7 times the odds (95% CI: 1.026, 13.425) of being willing to share biometric data via a wearable device paired to the DPS, compared to those who were less worried, after adjusting for other predictors. Participants with higher medical mistrust were less likely to be willing to share biometric data. For every one unit increase in medical mistrust score, the odds of not being willing to share biometric data via a wearable device increased by 0.8 (95% CI: 0.739, 0.946), after adjusting for other predictors. No significant association was found between the degree of substance use and willingness to share biometric data (p = 0.387; Table 2).

**Table 2:** Willingness to contribute digital phenotyping data and interact with ancillary systems in the context of DPS-based research (N = 131).

Outcome Variable *Willingness to…*	Exposure Variable	P- value	Beta Coefficient Estimates / SE	Measure of Association (OR) and 95% CI
Share biometric data	Daily PrEP Worry	0.046*	1.311 / 0.656	3.711 (1.026, 13.425)
	Degree of Substance Use	0.387	0.710 / 0.822	0.784 (0.313, 1.962)
	Medical Mistrust	0.005*	-0.179 / 0.063	0.836 (0.739, 0.946)
Share smartphone data	Daily PrEP Worry	0.006*	1.034 / 0.450	2.811 (1.163, 6.792)
	Degree of Substance Use	0.603	-0.243 / 0.468	0.784 (0.313, 1.962)
	Medical Mistrust	<0.0001*	-0.201 / 0.048	0.818 (0.745, 0.898)
Self-collect blood work	Daily PrEP Worry	0.225	0.517 / 0.426	1.677 (0.727, 3.865)
	Degree of Substance Use	0.483	-0.317 / 0.451	0.729 (0.301, 1.765)
	Medical Mistrust	0.001*	-0.139 / 0.043	0.870 (0.800, 0.947)
Receive text messages asking about substance use and sexual activity	Daily PrEP Worry	0.092	0.854 / 0.507	2.349 (0.870, 6.343)
	Degree of Substance Use	0.675	0.228 / 0.543	1.256 (0.433, 3.644)
	Medical Mistrust	<0.0001*	-0.273 / 0.058	0.757 (0.673, 0.851)
Receive text messages asking about general daily activities and location	Daily PrEP Worry	0.003*	1.307 / 0.441	3.693 (1.557, 8.763)
	Degree of Substance Use	0.828	-0.050 / 0.230	0.905 (0.368, 2.229)
	Medical Mistrust	0.0002*	-0.170 / 0.046	0.844 (0.770, 0.920)

*Statistically significant at the 0.05 level

### Willingness to share smartphone data

There was a statistically significant association between willingness to share smartphone data and both daily PrEP worry (p = 0.006) and medical mistrust **(**p <0.0001). Participants who reported worrying about daily PrEP adherence were more likely to be willing to share smartphone data, compared to those who were less worried, with an odds ratio of 2.811 (95% CI: 1.163, 6.792), after adjusting for other predictors. In addition, participants with higher medical mistrust were less likely to be willing to share smartphone data. For every one unit increase in medical mistrust, the odds of being willing to share smartphone data decreased by 20% (OR: 0.818; 95% CI: 0.745, 0.898), after adjusting for other predictors. No statistically significant association was found between the degree of substance use and willingness to share smartphone data (p = 0.603; Table 2).

### Willingness to participate in self-collected blood work

There was a statistically significant association between willingness to self-collect blood work and medical mistrust (p = 0.001). Participants with higher medical mistrust were less likely to be willing to self-collect blood work, with an odds ratio of 0.870 (95% CI: 0.800, 0.947), after adjusting for other predictors. No significant association was found between the degree of substance use or daily PrEP worry and willingness to self-collect blood work (p = 0.483 and p = 0.225, respectively; Table 2).

### Willingness to receive text messages asking about substance use, sexual activity, general daily activities, and location

#### Text messages asking about substance use and sexual activity

No statistically significant association was found between willingness to receive text messages asking about substance use and sexual activity, and daily PrEP worry (p = 0.092) or degree of substance use (p = 0.675). There was a statistically significant association between willingness to receive text messages asking about substance use and sexual activity, and medical mistrust (p <0.0001). For every one unit increase in medical mistrust score, the odds of being willing to receive text messages about substance use and sexual activity decreased by 0.76 (95% CI: 0.673, 0.851), after adjusting for other predictors (Table 2).

#### Text messages asking about general daily activities and location

There was a statistically significant association between willingness to receive text messages asking about daily activities and location, and both daily PrEP worry (p = 0.003) and medical mistrust (p = 0.0002). Participants who reported being worried or very worried about daily PrEP adherence had 3.7 times the odds of being willing to receive text messages asking about daily activities and location, compared to those who were not worried, after adjusting for other predictors (95% CI: 1.557, 8.763). Additionally, for every one unit increase in medical mistrust score, the odds of being willing to receive text messages asking about daily activities and location decreased by 17% (OR: 0.844; 95% CI: 0.770, 0.920), after adjusting for other predictors. No significant association was found between degree of substance use and willingness to receive text messages asking about daily activities and location (p = 0.828; Table 2).

## Discussion

Digital pills are evolving as a system to directly measure adherence to medications, including PrEP. Using DPS technology to better understand the contextual basis of PrEP adherence and nonadherence may help provide support to individuals who struggle with PrEP adherence at key junctures of risk. The emerging use of wearable devices and collection of smartphone-based digital phenotyping data may provide insight into key events where nonadherence is likely and the delivery of proactive, personalized adherence support may mitigate nonadherence [22–25]. Contrary to perceptions that individuals with substance use may be less accepting of the collection of personal data via mobile devices and other systems, the degree of substance use in our subsample was *not* associated the willingness of MSM on PrEP to interact with ancillary devices or text message-based queries to contextualize DPS-detected adherence data. We also found that participants who worried about their daily PrEP adherence and were more trusting of the medical system reported more willingness to contribute S1 Data–including biometric or digital phenotypic data from wearable devices, as well as self-collected blood samples–and to engage with text messages that query contextual behaviors linked to their PrEP adherence as measured by the DPS. These findings importantly frame the potential expansion of DPS technology through the integration of other wearable devices, self-collected biological samples, and the development of context-aware behavioral interventions. They also suggest opportunities to engage with community partners to address potential concerns related to medical mistrust around DPS technology and other, related systems for PrEP adherence measurement.

Overall, this subsample was also willing to contribute additional data to contextualize their adherence, despite their degree of self-reported substance use. This suggests that the addition of strategies like digital phenotyping or EMA surveys can add important context to observed PrEP adherence in MSM, and may present novel opportunities to teach and reinforce adherence skills in the setting of contextualized nonadherence behavior. Given the willingness of MSM to contribute smartphone-based data to further contextualize PrEP adherence behaviors, future work should focus on ethical, legal and social implications of smartphone data. Some potential strategies that address existing controversies in the ethics of digital phenotyping include responsible data collection strategies that only collect data that may be needed to understand contextual cues surrounding PrEP adherence, and design of security protocols that deidentify data, produce fuzziness in location data, and adequately explain the types of data collected to study participants. For example, clear explanation of the implications of location data and its relationship with PrEP adherence, substance use, and sexual activity should be disclosed to research participants, as well as, in the future, individuals who may leverage digital phenotyping in the context of their clinical care. Additionally, as PrEP initiation efforts continue to leverage telemedicine approaches to increase accessibility, the DPS may be an acceptable adherence measurement strategy that can be integrated into existing systems that already support self-collected biological samples for the assessment of sexually transmitted infections and regular HIV testing for PrEP users [26].

MSM in our subsample who reported more daily worry around PrEP adherence were significantly more likely to be willing to interact with text messages regarding their general daily activities and location that could be used to inform future adherence interventions. Participants’ increased willingness to share additional contextual data via ancillary devices suggests that many MSM may also be accepting of more personalized adherence interventions grounded in digital phenotypic measurements. Additionally, in research that integrates DPS-based PrEP adherence data to ground analysis of digital phenotyping data, MSM with substance use may be willing to respond to ecological momentary assessments that ask about sensitive and potentially stigmatizing sexual health, location and substance use. This suggests that future work may integrate strategies like text message-based queries and self-collected biological specimens into the DPS ecosystem to better understand PrEP adherence. As technologies like DPS and digital phenotyping are adopted, this work should remind researchers and policymakers that individuals with stigmatized conditions and substance use also can benefit and uptake these systems.

We also found that individuals who were more trusting of the medical system reported being significantly more willing to contribute additional digital phenotypic or contextual data in as part of DPS-based PrEP adherence research. A major challenge in light of these findings lies in decreasing the barriers to building participant/researcher, and ultimately patient/clinician, relationships that may improve overall trust in the medical system over time. Researchers may consider engaging with community partners or advocacy groups prior to the initiation of future studies in order to develop strategies for introducing the DPS to potential participants, and to adequately address concerns associated with trust in DPS technology and collection of phenotypic data from other systems. Such conversations should carefully consider the intersection of race, ethnicity, and existing levels of medical mistrust on users’ perceptions of the DPS and ancillary systems [27]. Our previous work suggests that the use of these monitoring technologies may, in fact, increase one’s sense of personal accountability for their PrEP adherence, as well as improve relationships with medical providers by providing objective data around PrEP ingestions, and the context in which they occur, to long-term primary care and sexual health services [10].

This investigation should motivate the continued development of digital health tools including behavioral interventions responding to medication adherence measured through various strategies including ingestible sensors. Importantly, future research should continue to include individuals with substance use disorder given their risk for HIV and other comorbidities. Research should also consider the role of care teams, including physicians, social workers, pharmacists, nurses and care coordinators in curating and responding to a suite of digital phenotyping and ingestible sensor data. Implementation challenges will include identifying the members of care teams who should receive context aware data. Existing care models that integrate a clinical pharmacist in adherence counseling as well as maintenance of the DPS (overencapsulation and technology teaching) may serve as a potential pathway to implementation of these systems. Integration of pharmacists into DPS infrastructure may also provide an implementation pathway in clinical settings with patient centered medical homes.

Digital phenotyping may also provide insights into how MSM with substance use experience challenges to adherence. These insights may then be translated into other populations (e.g., transgender individuals) and disease states (e.g., heart failure or diabetes medication adherence). In individuals with stigmatized conditions like their sexual orientation or risk factors including substance use, there may be a social desire to bias self-reported data in the context of research studies. Future development of digital phenotyping relying on native smartphone sensors may provide a more objective perspective to key behaviors that can be targets for empiric interventions that mitigate risk, reinforce adherence (in the setting of PrEP), and improve linkage to care. Future work may include observational studies to further characterize digital phenotypes that may be associated with substance use or its comorbidities, integration of digital phenotyping into adherence technologies like ingestible sensors, and research to develop predictive algorithms that present interventions at opportune moments to facilitation interaction with the user.

This study had several limitations. First, our sample size was recruited online from a social network site popular among MSM. As this recruitment strategy was selected to identify individuals with internet access, who were likely to have smartphones, and who engaged with social media platforms, this approach may have therefore missed key populations with different perspectives on DPS technology and ancillary systems. Participants who have internet access and most likely smartphones, may have higher levels of digital literacy, which could impact their willingness to contribute phenotypic data and engage with ancillary devices and systems. Second, the majority of participants identified as White MSM. Perceptions of PrEP, trust in the medical system, and experiences with digital health technologies may vary across race and ethnicity; as such, the conclusions drawn in this investigation may not be generalizable to non-White MSM [27]. The generalizability of the findings are also limited to the MSM community who engage in substance use. Third, individuals who are more concerned about their PrEP adherence and are more willing to share phenotypic data may be more interested in participating in research that focuses on these concerns, which may introduce sampling bias. Finally, this study involved a one-time quantitative assessment among *prospective* DPS users; participants did not have direct experience using the DPS but instead viewed a video describing its functionality and architecture prior to completing the quantitative assessment. Perceptions of and attitudes towards ancillary devices that contribute additional data to DPS-based PrEP adherence measures may be different following lived experience with the DPS.

## Conclusion

The DPS represents a unique opportunity for researchers, clinicians, and patients to better understand both PrEP adherence and nonadherence in the context in which it occurs. MSM with substance use may be accepting of DPS technology, willing to contribute digital phenotyping data, and willing to interact with ancillary systems in order to contextualize PrEP adherence patterns in a research setting. While substance use did not impact the willingness of MSM to accept these systems in this subsample, increased trust in the medical system and increased worry about daily PrEP adherence increased the likelihood that participants reported a willingness to interact with digital phenotyping, wearable devices, self-collected biological sampling, and text message queries to contextualize adherence.

## Supporting information

S1 Data Dataset with Codebook. (XLSX)
